# CIL-102-Induced Cell Cycle Arrest and Apoptosis in Colorectal Cancer Cells via Upregulation of p21 and GADD45

**DOI:** 10.1371/journal.pone.0168989

**Published:** 2017-01-09

**Authors:** Wen-Shih Huang, Yi-Hung Kuo, Hsing-Chun Kuo, Meng-Chiao Hsieh, Cheng-Yi Huang, Ko-Chao Lee, Kam-Fai Lee, Chien-Heng Shen, Shui-Yi Tung, Chih-Chuan Teng

**Affiliations:** 1 Division of Colon and Rectal Surgery, Department of Surgery, Chang Gung Memorial Hospital, Chiayi, Taiwan; 2 Chang Gung University College of Medicine, Taoyuan, Taiwan; 3 Graduate Institute of Clinical Medical Sciences, College of Medicine, Chang Gung University, Chiayi, Taiwan; 4 Department of Nursing, Chang Gung University of Science and Technology, Chiayi, Taiwan; 5 Research Center for Industry of Human Ecology and Research Center for Chinese Herbal Medicine, College of Human Ecology, Chang Gung University of Science and Technology, Taoyuan, Taiwan; 6 Chronic Diseases and Health Promotion Research Center, CGUST, Chiayi, Taiwan; 7 Division of Colorectal Surgery, Department of Surgery, Chang Gung Memorial Hospital, Kaohsiung Medical Center, Chang Gung University College of Medicine, Kaohsiung, Taiwan; 8 Department of Pathology, Chang Gung Memorial Hospital at Chiayi, Taiwan; 9 Department of Hepato-Gastroenterology, Chang Gung Memorial Hospital, Chiayi, Taiwan; University of South Alabama Mitchell Cancer Institute, UNITED STATES

## Abstract

CIL-102 (1-[4-(furo[2,3-b]quinolin-4-ylamino)phenyl]ethanone) is a well-known, major active agent of the alkaloid derivative of Camptotheca acuminata with valuable biological properties, including anti-tumorigenic activity. In this study, we investigated the molecular mechanisms by which CIL-102 mediated the induction of cell death, and we performed cell cycle G2/M arrest to clarify molecular changes in colorectal cancer cells (CRC). Treatment of DLD-1 cells with CIL-102 resulted in triggering the extrinsic apoptosis pathway through the activation of Fas-L, caspase-8 and the induction of Bid cleavage and cytochrome c release in a time-dependent manner. In addition, CIL-102 mediated apoptosis and G2/M arrest by phosphorylation of the Jun N-terminus kinase (JNK1/2) signaling pathway. This resulted in the expression of NFκB p50, p300 and CREB-binding protein (CBP) levels, and in the induction of p21 and GADD45 as well as the decreased association of cdc2/cyclin B. Furthermore, treatment with the JNK1/2 (SP600125), NFκB (PDTI) or the p300/CBP (C646) inhibitors abolished CIL-102-induced cell cycle G2/M arrest and reversed the association of cdc2 with cyclin B. Therefore, we demonstrated that there was an increase in the cellular levels of p21 and GADD45 by CIL-102 reduction in cell viability and cell cycle arrest via the activation of the JNK1/2, NFκB p50, p300 and CBP signaling modules. Collectively, our results demonstrated that CIL-102 induced cell cycle arrest and apoptosis of colon cancer cells by upregulating p21 and GADD45 expression and by activating JNK1/2, NFκB p50 and p300 to provide a new mechanism for CIL-102 treatment.

## Introduction

Colorectal cancer (CRC), an aggressive malignant disease with a poor prognosis, is the fourth leading cause of cancer-related death in the industrialized world [1]. A large body of evidence indicates CRC cells’ self-sufficiency in growth signals, their ability to escape from apoptosis, and their tendency toward tissue invasion and metastasis [2]. Moreover, chemotherapy treatments for CRC are often ineffective because of the intrinsic chemoresistance of these tumors [3]. Therefore, it is imperative to develop more effective drugs. Apoptosis is a morphologically and biochemically driven process, while impaired apoptosis and defects in the regulation of the cell cycle are hallmarks that contribute to cancer growth and aggressiveness [4]. Recent studies have suggested that phenolic phytochemicals having antioxidant activity should short-circuit the signaling events and eventually inhibit CRC cell proliferation [5]. Previous study has shown that Camptothecin (CPT) is an alkaloid originally isolated from the bark and stem of *Camptotheca acuminata*, a tree native to China [6]. As described in detail previously that CPT induces cancer cells apoptosis, interacts with DNA to form a complex, and reduces DNA, RNA, and protein synthesis [7]. A number of furo[2,3-b]quinoline derivatives, such as CIL-102, have also been synthesized and have been found to exhibit antitumor effects [8]. The survival curve showed that CIL-102 may possess antitumor activity against cancer cells of the prostate and breast, as well as those found in leukemia and cervical carcinoma [9]. This is studied by the inhibition of tubulin polymerization followed by apoptosis processed via the caspase and non-caspase pathways [10]. However, little is known about the anti-colorectal cancer mechanism. Similarly, the mechanism by which CIL-102 initiates cell death and cell cycle arrest and by which the signaling cascades become activated remains poorly understood.

Chemopreventive compounds may induce cell apoptosis through the regulation of the MAPK pathways [11]. The mitogen-activated protein kinases (MAPKs) are a family of protein kinases that transfer signals of stimuli from the cell membrane to the nucleus [12]. Intracellular signaling pathways for apoptosis have mainly focused on two cascades, the extrinsic and intrinsic pathways, which both lead to the kinase cascade [13]. The extrinsic pathway is triggered at the plasma membrane by the activation of TNFR1/Fas. This results in the cleavage of cytosolic BID to truncated tBID by caspase-8, which translocates to the mitochondria [14]. The kinase-signaling cascade, the intrinsic pathway, was originally identified as an important pathway in the transduction of apoptotic signals initiated by stress or anticancer drug stimuli [15]. The release of cytochrome c from the mitochondria represents a critical event in initiating the activation of caspase 9 and caspase 3 [16]. Herein, the effect of CIL-102-mediated apoptosis and the triggering mechanism of the signaling pathways on human CRC remain unclear.

Proliferation of CRC cells usually fails to control checkpoints, one of which is the G2/M phase checkpoint [17]. The inhibitors of the cell cycle progression stimulatory signaling pathway have been shown to target cdc2, cyclin B1 and cyclin A, which are required for mitotic entry in mammalian cells, thereby suppressing cdc2 activity by inhibiting the accumulation of cyclin B1 mRNA and protein [18]. DNA damage also correlates with the inhibition of cdc2-cyclin B kinase activity through clinical treatment with anti-microtubule drugs to inhibit colorectal cells’ proliferation and induce cell growth arrest [10, 17]. A cyclin-dependent kinase inhibitor called p21/WAF1 plays an important role as a tumor suppressor [19]. With respect to cell-cycle regulation, p21 has been reported to induce G2/M arrest by blocking CDK1 activity. It was suggested that p21/WAF1 and Gadd45 may participate in a coordinated manner in a DNA damage response [20]. Evidence has emerged that GADD45, a cell cycle regulated nuclear protein, disrupts the interactions of Cdc2 with cyclin B1 and that GADD45 may thus induce G2/M arrest. Therefore, it is imperative to investigate how diet prevents the malignance and progression of CRC [21]. In addition to previous strategies that can reduce CRC risk, dietary phytochemicals are emerging as a promising chemoprevention approach. The cell cycle regulation and the *in vivo* anti-tumor effect of the 9-anilinofuroquinoline derivative, CIL-102, are not clearly known in CRC. GADD45 and p21, therefore, may represent a unique target for drugs that induce cell cycle arrest, apoptosis, and differentiation such as CIL-102.

The 9-anilinofuroquinoline derivative, CIL-102, has been used clinically as an antiseptic drug, which was not a natural product and, is impossible to be found in the bark and stem of Camptotheca acuminate [22]. Numerous studies have suggested that it possesses anticancer and chemopreventive properties and inhibits the proliferation of tumor cells [23, 24]. Our recent study showed that CIL-102 inhibited the proliferation and the invasiveness property in glioma cells and altered the expression of genes related to cell cycle regulation by activating the ERK1/2 and Cdc25cSer^216^ cell-cycle-related proteins and inducing ROS generation [23]. However, the mechanism by which CIL-102 induces apoptosis remains poorly understood. In our study, we first investigated whether CIL-102 had a dose-dependent effect on the cytotoxicity of CRC. It was found to cause apoptosis, which was preceded by the sustained activation of JNK, activated caspase-8 and cleaved Bid protein to its truncated form, t-Bid, and caused the release of cytochrome c. It then directly activated the downstream effector caspases such as caspase-3 and caspase-9. Our results strongly suggested an essential role for the JNK1/2/NFκB p50/p300/CBP as well as the p21 and GADD45 pathways during the execution of cell cycle G2/M arrest, which might be controlled by inhibiting CRC cell proliferation and which seems to play a role in CIL-102-induced apoptosis.

## Materials and Methods

### Chemical reagents and antibodies

All culture materials were purchased from Gibco (Grand Island, NY, USA). 1-[4-(Furo[2,3-b]quinolin-4-ylamino)phenyl]ethanone (CIL-102), 3-(4,5-dimethylthiazol-2-yl)-2,5-diphenyltetrazolium bromide (MTT), ROS scavenger (*N*-acetyl cysteine [NAC]), 2,7-dichlorodihydrofluorescein diacetate (H_2_DCFDA), dihydroethidium (DHE), ERK inhibitor (PD98059), c-Jun N-terminal kinase (JNK1/2) inhibitor (SP600125), p38 inhibitor (SB203580), and mTOR inhibitor (rapamycin) were purchased from Sigma (St. Louis, MO, USA). Mouse monoclonal antibodies against cyclin A, cyclin D1, cyclin E, cyclin B1, acetylation of H3 (Ac-Histone H3) at Lys 9 and Lys 14, cytochrome c, caspase-3, -8, -9 and β-actin were purchased from Santa Cruz Biotechnology (Santa Cruz, CA, USA). Rabbit polyclonal antibodies against cdk2, ERK1/2Thr^202^Ty^r204^, p38 Thr^180^Tyr^182^, and JNK1/2 Thr^183^Tyr^185^ mouse monoclonal cdc2, t-BID, p300, CBP, p21, GADD45, and Fas-L antibodies were purchased from Cell Signaling Technology (Beverly, MA, USA). SDS, NP-40, sodium deoxycholate, protease inhibitor cocktails were purchased from Sigma (St Louis, MO, USA).

### Cell culture

Human colon cancer cell line DLD-1 (CCL-221) and human colorectal carcinoma cell line HCT-116 (CCL-247) were purchased from American Type Culture Collection (ATCC). DLD-1 cells were cultured in RPMI 1640 medium composed of 10% fetal calf serum (FCS) (S0113; Biochrom KG, Berlin, Germany) and 1% antibiotics (100 units/mL of penicillin and 100 μg/mL of streptomycin) (Sigma Chemicals, St. Louis, MO, USA) and incubated at 37°C with 5% CO2. We purchased passage number 1 of human normal astrocytes (HNA) from ScienCell Research Laboratories (Carlsbad, CA) and cells were grown. Adhered cells were washed twice with PBS. HCT-116 was cultured in DMEM supplemented with 10% heat-inactivated newborn calf serum at 37°C in a humidified 5% CO2 incubator [25].

### Cell growth and proliferation assay

The previously reported MTT quantitative colorimetric assay was previously described [23,26]. Cells were seeded and incubated with the various agents. Thereafter, the medium was changed, and cells were incubated with MTT (0.5 mg/mL) for 4 h. The viable cell number was directly proportional to the production of formazan, which was measured spectrophotometrically (λ = 563 nm) after solubilization with isopropanol. Cell growth was determined by counting the cells at the indicated time points with a Coulter counter, combined with a trypan blue (0.2%) exclusion assay [27].

### Apoptosis assay and cell cycle distribution analysis

Changes in cell morphological characteristics during apoptosis were examined using fluorescence microscopy of 4′,6-diamidino-2-phenylindole (DAPI)-stained cells, as described in detail previously [23,27]. The monolayer of cells was fixed with 4% paraformaldehyde for 30 min at room temperature. The fixed cells were permeabilized with 3 treatments in 0.2% Triton X-100 in phosphate-buffered saline, followed by incubation with 1 μg/mL of DAPI for 30 min. The apoptotic nuclei were detected under 200× magnification using a fluorescent microscope with a 340/380 nm excitation filter and were scored according to the percentage of apoptotic nuclei found in samples containing 200 to 300 cells.

Cell viability, as previously reported by Annexin V–FITC/ propidium iodide (Biosource International, USA), used to quantify the percentage of cells undergoing apoptosis. and the cells were washed prior to FACS analysis and Cell Quest software was used (Becton Dickenson). The results were presented as a percentage of the fluorescent intensity compared with the control sample. Data were analyzed with CellQuest and WinMDI software. The apoptotic cells (V+/PI-) were measured by the fluorescence-activated cell sorter analysis in a FACS analyzer (Becton Dickinson). The data represented three independent experiments [26].

Cell-cycle distribution was analyzed using flow cytometry. Cells stained with propidium iodide were analyzed with a FACScalibur™ (Becton Dickinson), and the data were analyzed using a mod-fit cell cycle analysis program [26].

### Preparation of total cell extracts and immunoblot analyses

Cells were lysed with a buffer containing 1% NP-40, 0.5% sodium deoxycholate, 0.1% sodium dodecyl sulfate (SDS), and a protease inhibitor mixture (phenylmethylsulfonyl fluoride, aprotinin, and sodium orthovanadate). The total cell lysate (50 μg of protein) was separated by SDS-polyacrylamide gel electrophoresis (PAGE) (12% running, 4% stacking) and analyzed by using the designated antibodies and the Western-Light chemiluminescent detection system (Bio-Rad, Hercules, CA), as previously described [23,28].

### Statistical analysis

The experiments were performed in triplicate and data were reported as the mean ± standard deviation from 3 independent experiments and evaluated by one-way ANOVA. Significant differences were established at *P* < 0.05 [28].

## Results

### Effects of CIL-102 on the viability of human CRC cells

**Fig 1:**
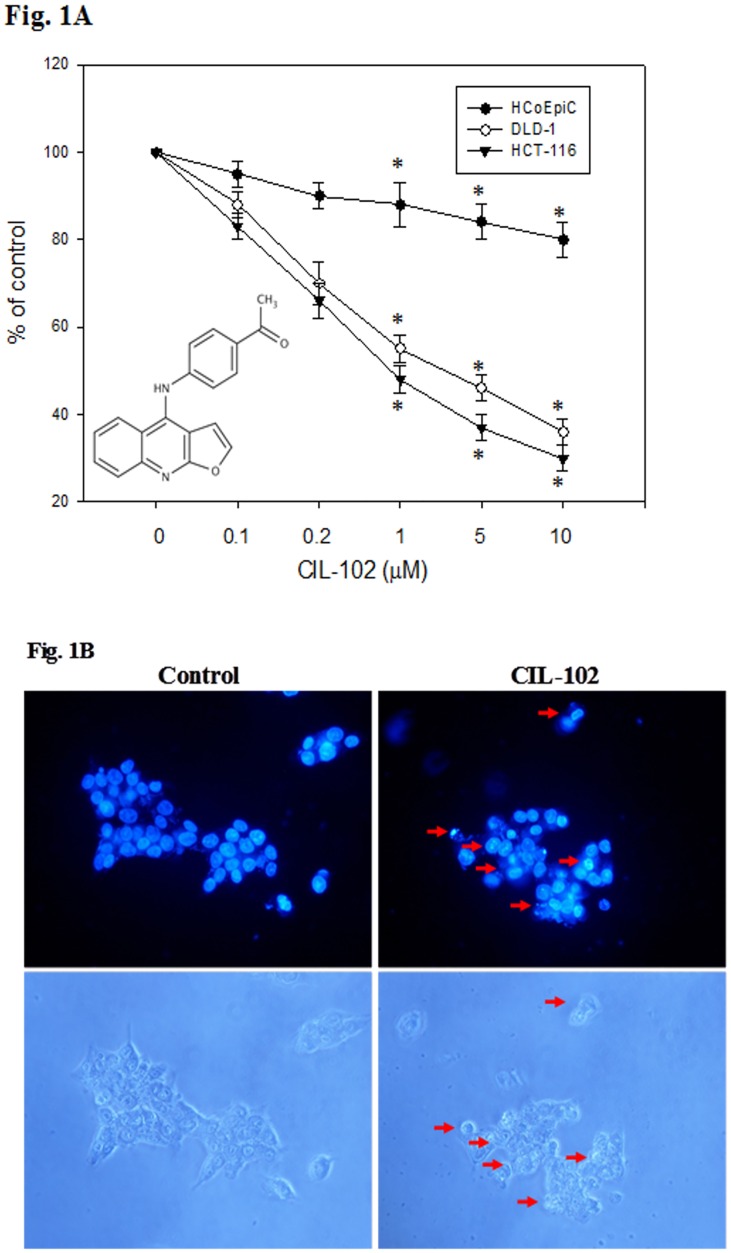
Effect of CIL-102 on cell viability and morphological characteristics of human colorectal cancer DLD-1 cells, and its role in assessing cell death. (A) DLD-1, HAT-116 and HCoEpiC cell were treated with either 0.1% DMSO (as control) or CIL-102 (0.1–10 mM) for 24 h and the proportion of surviving cells was measured by the MTT assay. (B) Changes in nuclei by DAPI staining. DLD-1 cells were treated with vehicle or 1 mM CIL-102 for 24 h, and stained with DAPI. Apoptotic cells were measured under fluorescence microscopy. The data were presented as the mean of three repeats from one independent experiment. Other data in this figure is presented as mean ± SD of three independent experiments. * indicate means that are significantly different when compared to the control group of DLD-1 with P < 0.05.

By evaluating the apoptosis and anti-invasion potential involving the signaling pathway, we assayed whether CIL-102 provides substantial therapeutic advantages. To determine whether CIL-102 is cytotoxic to human CRC cells, we evaluated the apoptosis and anti-tumor proliferation potential involving the signaling pathway. We treated DLD-1, HCT-116 and normal human colonic epithelial cells (HCoEpiC) with a range of CIL-102 doses for 24 h and examined them by MTT assays. CIL-102 treatment resulted in a dose-dependent loss of cell viability, as shown in Fig 1A. After treatment with 1 μM CIL-102 for 24 h, 55% and 50% of DLD-1 and HCT-116 cells (*P* < 0.01), respectively, survived in culture (Fig 1A). However, CIL-102 did not significantly show cytotoxic effects in HCoEpiC cells. In addition, to verify CIL-102-induced cell toxicity, we examined the changes in cell morphology after CIL-102 exposure. Fig 1B shows that exposure to erinacine A for 24 h caused DLD-1 cells to morphologically display features of cell shrinking, with the growths to which they belong becoming smaller.

### CIL-102 induces apoptosis and cell cycle G2/M arrest in DLD-1 CRC cells

**Fig 2:**
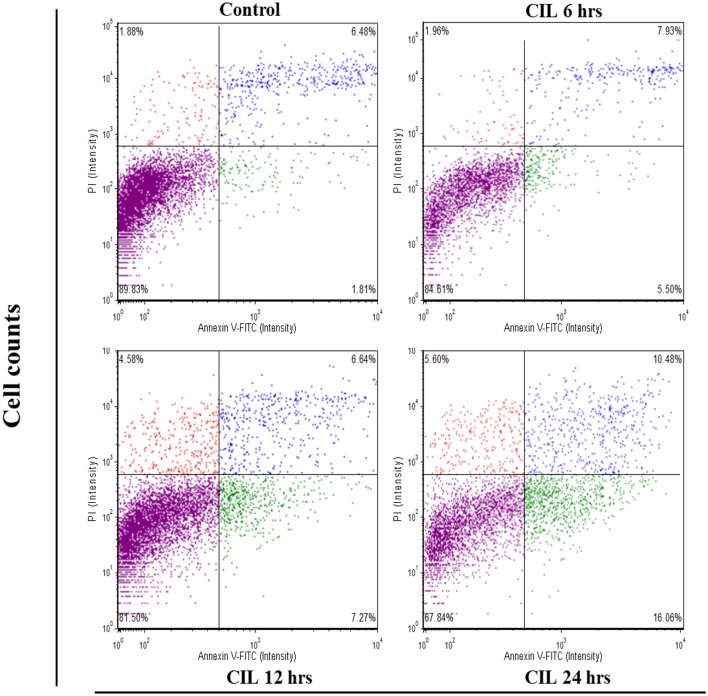
After an indicated treatment for 24 h, the DLD-1 cells were stained with FITC-conjugated Annexin-V and PI for flow cytometry analysis as described in Materials and methods. The percentages presented in each frame depicted the apoptotic cells. *P <0.01, compared with the control group (0.2% DMSO).

**Fig 3:**
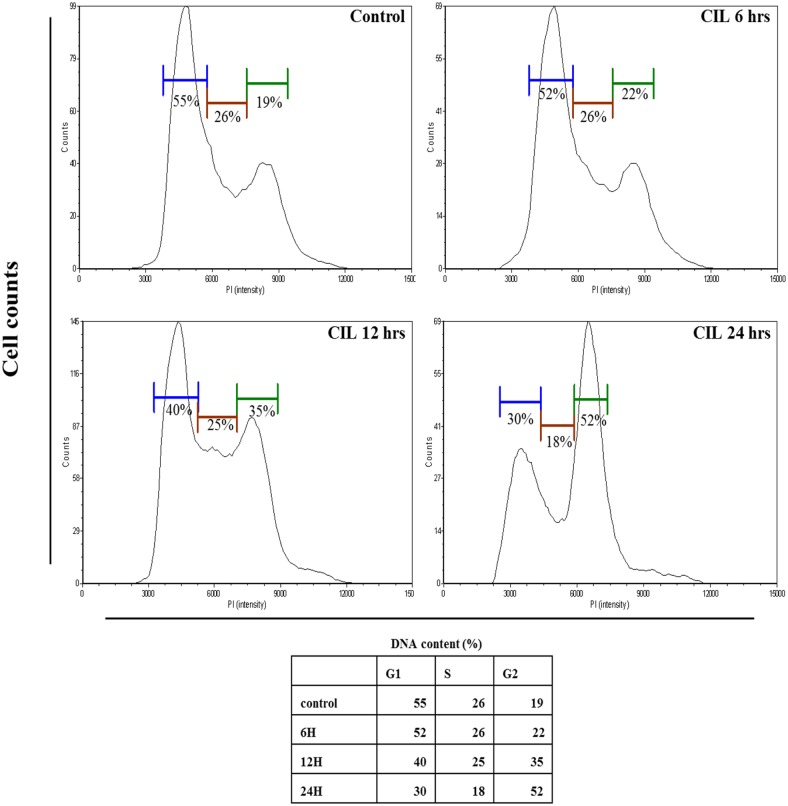
Effect of CIL-102 on cell cycle distribution in DLD-1 cells. (A) After treatment with Erinacine A (1 mM) for 24 h, the cells were fixed and stained with propidium iodide, and the DNA content was analyzed by flow cytometry (FACS). The cell number percentage in each phase (G1, S, and G2/M) of cell cycle was calculated and expressed.

The addition of Annexin-V stain has led to similar findings (Fig 2). The extent of apoptosis of CIL-102 induction was quantified as a percentage of annexin V-positive cells and was shown as 12 ± 4 (6 h), 13 ±2 (12 h), and 26 ± 3% (24 h), respectively. Both experiments proved that CIL-102 elicits apoptosis. Cell cycle distribution analysis showed that CIL-102 induces cell cycle G2/M arrest in DLD-1 cells. As demonstrated in Fig 3, following a 24 h treatment with 1 μM CIL-102, the percentages of G2/M phase cells increased to 22 ± 2 (6 h), 35 ±2 (12 h), and 52 ± 2% (24 h), respectively. These results suggest that CIL-102 induces G2/M arrest in a time-dependent manner.

### Activation of apoptosis pathway by CIL-102 in DLD-1 cells

**Fig 4:**
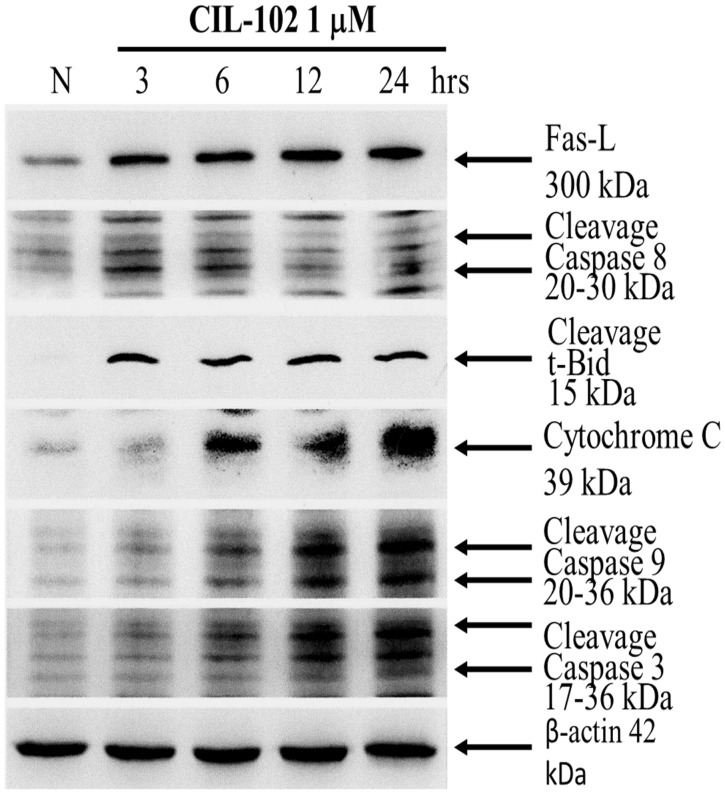
Effect of CIL-102 on Fas-L, caspase 8, t-Bid, cytochrome c, caspase 3 and caspase 9. Cells were treated with CIL-102 for 0–24 hr, and were separated by a 15% SDS–PAGE, and, subsequently, immunoblotted with antibodies against Fas-L, cleavage caspase 8, cleavage t-Bid, cytochrome c, cleavage caspase 3 and cleavage caspase 9, or b-actin which served as internal control. CIL-102 induced translocation of cytochrome c. Equal amounts of protein from cytosolic fraction of DLD-1 cells which has been treated with 1 mM of CIL-102.

Recent theories pertain to the persistent extrinsic apoptosis pathway involved in the binding of a ligand to one of the tumor necrosis factor families of death receptors, followed by the activation of caspase-8 by phytochemicals. It was concluded that the intrinsic pathways are linked through the ability of caspase-8 to cleave Bid, which in turn leads to the release of cytochrome c from the mitochondria pathways. To elucidate the events of CIL-102-stimulated DLD-1 cell death, we examined kinetic studies to evaluate Fas-L, caspase 8 and t-BID expression as well as caspase 3 and caspase 9 during a 24 h period. As shown in Fig 4, CIL-102 was shown to cause detectable and increased levels of Fas-L and cytochrome c in the cytosol and a time-dependent cleavage of Bid for 24 h. Treatment with CIL-102 resulted in proteolytic processing of the caspase 3 and caspase 9 (36 kDa) into two smaller subunits.

### Expression of p21 and GADD45 as well as NFκB p50/p300/CBP signaling pathways by CIL-102 in DLD-1 cells

**Fig 5:**
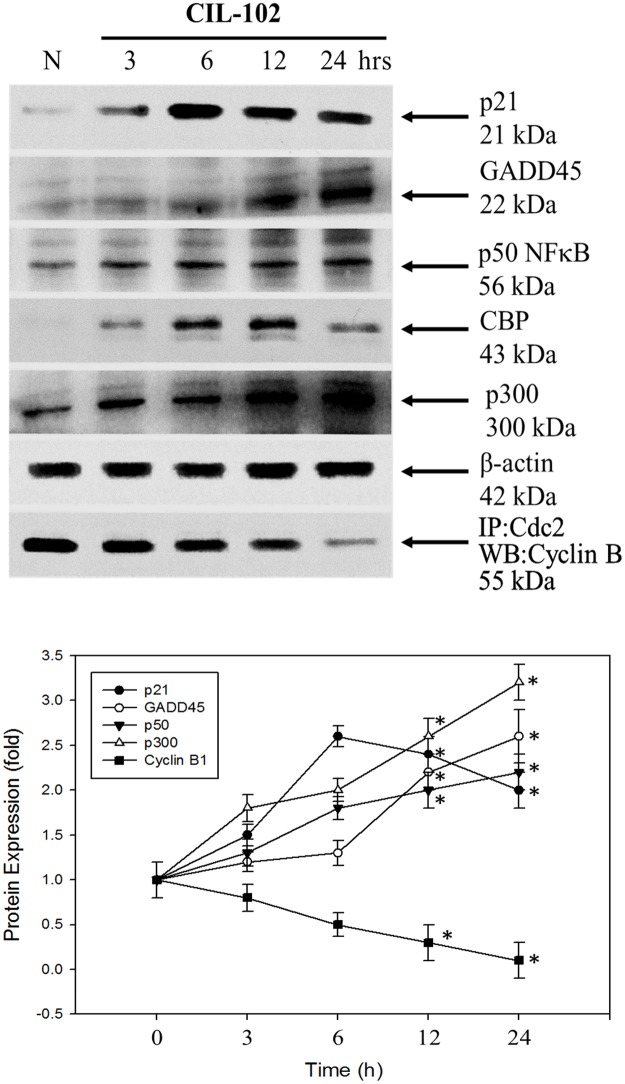
Effect of CIL-102 on the p21, GADD45, p50 NFκB, p300/CBP, CREB as well as expression of cell cycle associated proteins. DLD-1 cells were pretreated with CIL-102 for indicated time points. Whole cell lysate proteins were prepared and analyzed by western blot, with β-actin serving as loading control. CIL-102 for the indicated times were analyzed by 10% SDS-PAGE and subsequently treated with antibodies against p21, GADD45, p50 NFκB, p300/CBP and CREB. The association of cdc2 with Cyclin B were determined by immunoprecipitation followed by western blot with antibody.

Numerous investigations have shown the among proteins known to the transcriptional co-activator p300/CBP deserves DNA damage that control G2/M regulation and delayed entry into mitosis correlation with cdc2/cyclin B1 association [29]. To demonstrate the effect of CIL-102 on the cell-cycle-related proteins and the kinase-signaling pathway, we assessed whole-cell lysates from erinacine A–treated DLD-1 cells by Western blot analysis using antibodies against the expressed forms of p21 and GADD45 as well as NFκB p50/p300/CBP. As shown in Fig 5, treatment of DLD-1 cells with CIL-102 resulted in the marked expression of p21 and GADD45 as well as NFκB p50/p300/CBP compared with the control for a period of time points. In addition, the analysis showed a marked downregulation of cdc2/cyclin B1 association after CIL-102 treatment of DLD-1 cells in a time-dependent manner.

### JNK1/2 MAPK and p50/p300 pathways are involved in the regulation of CIL-102-induced cell death and cell cycle G2/M arrest in DLD-1 cells

**Fig 6:**
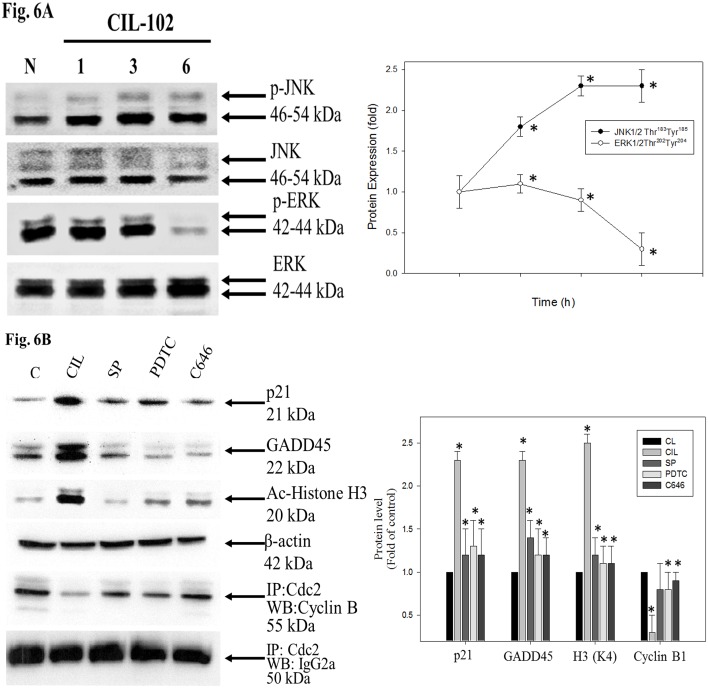
Effect of the kinase inhibitors in blocking CIL-102-induced cell cycle G2/M arrest and apoptosis-related protein. (A) The expression levels of phosphorylation and protein levels of JNK and ERK, after treatment with CIL-102. DLD-1 cells were treated with CIL-102 for the indicated time points. Total cell lysates were analyzed by SDS-PAGE and subsequently immunoblotted with antisera. (B) DLD-1 were incubated with various concentrations of the specific JNK1/2 inhibitor SP600125, NFκB inhibitor PDTI or the p300/CREB-Binding Protein inhibitor C646 for 1 h. Next, the cells were treated with CIL-102 for 12 h. Total cell lysates of cells treated with or without CIL-102 for the indicated time were extracted, and the expression of p21, GADD45, p50 NFκB, Histone H3 (K4) as well as The association of cdc2 with Cyclin B were determined, as described in “Materials and methods”. Protein levels were quantified by densitometric analysis with the ratio of untreated control being set 1 fold. The quantitative data were presented as the mean of three repeats from one independent experiment. The data were presented as mean ± SD of three independent experiments. *P<0.05, compared to the control group.

Western blot analysis was used to determine whether CIL-102 induces apoptosis and cell cycle arrest by modulating the level of cell-cycle-related proteins, as shown in Fig 6A. The analysis showed a marked expression, with the treatment of DLD-1 cells with CIL-102 resulting in a time-dependent phosphorylation of JNK1/2 Thr^183^183/Tyr^185^ at both 3 h and 6 h. Activation of both kinases was observed after treatment with CIL-102 and was markedly sustained for at least 6 h. ERK1/2Thr^202^/Tyr^204^ was found to be not constitutively activated, but it exhibited no changes after CIL-102 treatment. To investigate the roles of the JNK1/2 signaling pathway and the NFκB p50/p300 pathways in CIL-102-induced apoptosis and cell cycle retardation, we exposed DLD-1 cells to CIL-102 and then co-treated them with the specific JNK inhibitor SP600125, NF-κB p50 inhibitor (PDTC), or the p300/CBP inhibitor C646. The effects of those inhibitors in blocking CIL-102-induced cell death were assessed, and the cell cycle G2M distribution (%) was determined. As shown in Table 1, JNK inhibitor SP600125, NF-κB p50 inhibitor (PDTC), and p300/CBP inhibitor C646 almost blocked CIL-induced cell death and cell cycle G2/M arrest in DLD-1 cells. Furthermore, it was found that the expression of these proteins, p21 and GADD45, and the acetylation of H3 (Ac-Histone H3) at Lys 9 and Lys 14 clearly increased with the use of CIL-102, compared with the control. This data significantly demonstrated that CIL-102 inhibited cell growth and decreased the association of cdc2 with cyclin B (Fig 6B). We then examined the regulation of the JNK1/2 MAPK and p50/p300 pathways in CIL-102-mediated DLD-1 cell cycle arrest, while cells were incubated for 2 h with the specific inhibitors JNK inhibitor SP600125, NF-κB p50 inhibitor (PDTC) and p300/CBP inhibitor C646, and were then treated with CIL-102. The expressions of p21, GADD45, and Ac-Histone H3, as well as the association of cdc2/cyclin B, were analyzed. SP600125, PDTC and C646 significantly reversed the CIL-102–induced expression of p21, GADD45, Ac-Histone H3 and the reduced association of cdc2/cyclin B. Taken together, these results show that CIL-102 induces sustained activation of the JNK1/2 and p50/p300 pathway, GADD45 and p21, and that the apoptotic pathway is required for CIL-102 inhibition of DLD-1 cell growth (Fig 6B).

**Table 1:** Effects of the kinase inhibitor on the CIL-102 treatment associated with apoptosis and cell cycle distribution in DLD-1 cells.

	% of cell death	Cell cycle G2M arrest (%)
**Control**	**6**	**18**
**CIL-102**	**28**	**54**
**CIL-102+SP**	**14**	**34**
**CIL-102+PDTC**	**13**	**30**
**CIL-102+C646**	**12**	**26**

## Discussion

The development of colorectal cancer (CRC) is a sequential multistage process consisting of tumor initiation, tumor promotion, and tumor metastasis [30]. Despite intense efforts to develop treatments, effective agents have not yet been found. Medicinal herbs or foods are a potential source of chemopreventive compounds for antitumor activities that target the apoptosis pathways in cancer cells that damage DNA and activate the signaling pathways, and for the expression of proteins involved in the growth arrest of cancer cells [5, 11]. Previous studies have shown that anticancer drugs, including Cisplatin, Camptothecin, Etoposide and Doxorubincin, act by inflicting DNA damage. Most of these bioactive compounds are plant secondary metabolites such as terpenoids, flavonoids and alkaloids, all of which kill tumor cells by apoptosis induction [31]. Our *in vitro* data demonstrated that furo[2,3-b]quinoline derivatives, such as CIL-102, and treatment above a concentration of 1 μM resulted in a significant antiproliferative effect against human colorectal cancer cells but not for normal HCoEpiC cells (Fig 1). Here, CIL-102 treatment at the concentration of 10 μM for 24 h resulted in an induction of DLD-1 cell apoptosis (Fig 2). We also found that CIL-102 induced DLD-1 cell accumulation in the G2/M phase (Fig 3), the same as the effect on other HCT-116 cells (unpublished data). CIL-102 was studied for the mechanism of its action in the apoptotic pathway in the human colonic carcinoma cell. Treatment of CIL-102 resulted in the activation of the caspase 8, caspase 9 and caspase 3, and the release of cytochrome c in the time-dependent induction of apoptosis (Fig 4). In addition, there was an increase in the cellular levels of phospho JNK1/2, Fas-L and t-BID in the CIL-102-induced apoptosis via the activation of JNK (Figs 4 and 6A). The induction of apoptosis was alleviated by inhibitors for JNK1/2MAPK (Table 1). Furthermore, our study demonstrated that the cytotoxic effect of CIL-102 on the DLD-1 appeared to be through G2/M phase arrest with GADD45 and p21 expression, as well as activation of the p50/p300/CBP pathway, and that it caused a decrease in cd2/cyclin B1 activity (Fig 5). Additionally, the JNK1/2 and p50/p300 pathways were directly involved in activating GADD45 and p21 during the mitosis transition (Table 1). CIL-102 activated JNK1/2 and p50/p300 as well as the expression of GADD45 and p21 on DLD-1 following the DNA damage response, suggesting this as a target with CIL-102 (Fig 6B).

Studies have shown that the potential antitumor activity of CIL-102 may be mediated mitotic arrest and apoptosis in human prostate cancer cells via binding to tubulin and disrupting microtubule organization with a variety of signaling pathways [10]. Our previous study demonstrated that CIL-102 inhibited proliferation in human astrocytoma cells by activating the ERK1/2 and Cdc25cSer^216^ cell-cycle-related proteins and inducing ROS production, leading to oxidative stress [23, 24]. Therefore, based on this study, by evaluating the apoptosis and mitotic phase arrest involving the signaling pathway, we assayed whether CIL-102 related to growth inhibition and activation of the signaling pathway in human CRC cells provides substantial therapeutic advantages. Recent findings have demonstrated that the overexpression of Gadd45 in human normal fibroblasts causes cells to arrest in the mitotic phase. Cancer cells show abnormality of the cell cycle G2/M checkpoint following exposure to certain DNA-damaging agents [32]. Gadd45 has been shown to interact with cdc2 kinase, which is required for the G2-M transition. The interaction between GADD45 and p21 results in the inhibition of cdc2-cyclin B1 kinase activity [20, 32]. Our results determined that the upregulation of GADD45 and p21 protein affected the G2/M cell cycle arrest of DLD-1 cells with a decrease in the association of the cdc2/cyclin B1 complexes, which was preceded by the JNK1/2MAPK/NF-κBp50/p300/CBP pathway (Fig 6).

Many studies have shown that cellular mechanisms contribute to the overall cancer-prevention effects of these dietary phytochemicals [33]. Natural phytochemicals from certain plants have the capability to affect the epigenome and can also trigger sustained DNA damage and apoptosis induction. Additionally, they can disrupt the G2/M cell cycle in cancer cells exposed to dietary phytochemicals such as histone deacetylase inhibitors (HDAC) [34]. Inhibition of HDAC activity may occur in human colon cancer lines, with an increase in histone H3 acetylation in global or local histone modification status, such as induction of TRAIL, DR4 and Fas-L, p21 genes, via histone acetyltransferase (HAT) p300/CBP [35, 36]. Furthermore, the current *in vitro* study demonstrated that CIL-102 treatment significantly upregulated the expression of p300/CBP and acetylation of H3 (Ac-Histone H3) in DLD-1 cells (Figs 5 and 6B). Thus, CIL-102, as an individual dietary phytochemical, represents a novel chemotherapeutic agent worth continued investigation in the treatment of CRC. Additional studies are still needed to elucidate the CIL-102 effects on the HAT and HDAC between different molecular cellular signaling pathways and epigenetic machinery as well as to determine *in vivo* CRC cells’ xenograft and CIL-102 dose treatment.

**Fig 7:**
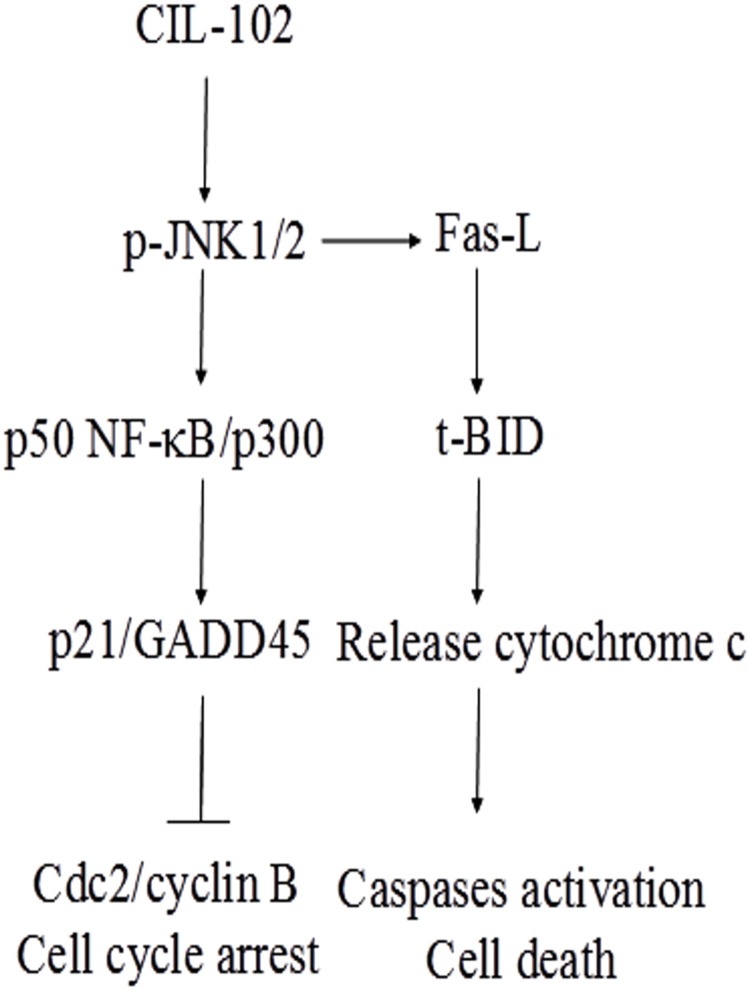
Schematic presentation of the signaling pathways involved in CIL-102 induced cell cycle arrest G2/M phase and apoptosis in human DLD-1 cancer cells. The effect of CIL-102 on the activation JNK1/2, and p50 NF-κB/p300 pathways, which leads to p21 and GADD45 expression and cdc2/cyclin B inactivation and increases cell cycle arrest in human DLD-1. CIL-102 triggered the extrinsic apoptosis pathway through activation of Fas-L and induction of Bid cleavage, cytochrome c release and caspase-8, -3, -9 activation.

In this study, we evaluated CIL-102-induced apoptosis and the cell cycle arrest G2/M phase in human DLD-1 cancer cells as a result of the expression of p21 and GADD45 and cdc2/cyclin B inactivation by the activation of the JNK1/2 signaling pathway and the p50NF-κB/p300/CBP pathway. These results lead us to speculate that CIL-102 may play a role in an apoptotic cascade in DLD-1 cells via Fas-L, t-BID expression and cytochrome c release, and caspase-8, 3, and 9 activation (Fig 7). This study is potentially interesting with regard to the antitumor effect of CIL-102 as it relates to the development of novel chemotherapeutic approaches in the treatment of malignant CRC. Further study is required.

## References

[1] Haggar FA, Boushey RP. Colorectal cancer epidemiology: incidence, mortality, survival, and risk factors. Clin Colon Rectal Surg. 2009; 22:191–7. 10.1055/s-0029-1242458 21037809 PMC2796096

[2] Sillars-Hardebol AH, Carvalho B, de Wit M, Postma C, Delis-van Diemen PM, Mongera S et al Identification of key genes for carcinogenic pathways associated with colorectal adenoma-to-carcinoma progression. Tumour Biol. 2010; 31:89–96. 10.1007/s13277-009-0012-1 20358421 PMC2848338

[3] Chan SK, Griffith OL, Tai IT, Jones SJ. Meta-analysis of colorectal cancer gene expression profiling studies identifies consistently reported candidate biomarkers. Cancer Epidemiol Biomarkers Prev. 2008; 17:543–52. 10.1158/1055-9965.EPI-07-2615 18349271

[4] Hanahan D, Weinberg RA. The hallmarks of cancer. Cell. 2000; 100:57–70. 10647931 10.1016/s0092-8674(00)81683-9

[5] Li-Weber M. Targeting apoptosis pathways in cancer by Chinese medicine. Cancer Lett. 2013; 332:304–12. 10.1016/j.canlet.2010.07.015 20685036

[6] Chen IL, Chen YL, Tzeng CC, Tzeng CC, Chen IS. Synthesis and cytotoxic evaluation of some 4-anilinofuro[2,3-b]quinoline derivatives. Helv Chim Acta 2002; 85:2214–21.

[7] Chen IL, Chen YL, Tzeng CC. An efficient synthesis of antitumor 4-anilinofuro [2,3-b]quinoline derivatives. Chin Pharm J. 2003; 55:49–53.

[8] Zhao YL, Chen YL, Tzeng CC, Chen IL, Wang TC, Han CH. Synthesis and cytotoxic evaluation of certain 4-(phenylamino)furo[2,3-b]quinoline and 2-(furan-2-yl)-4-(phenylamino)quinoline derivatives. Chem Biodivers. 2005; 2:205–14. 10.1002/cbdv.200590003 17191973

[9] Chen YL, Chen IL, Wang TC, Han CH, Tzeng CC. Synthesis and anticancer evaluation of certain 4-anilinofuro[2,3-b]quinoline and 4-anilinofuro[3,2-c]quinolone derivatives. Eur J Med Chem. 2005; 40:928–34. 10.1016/j.ejmech.2005.04.003 15913847

[10] Huang YT, Huang DM, Guh JH, Chen IL, Tzeng CC, Teng CM. CIL-102 interacts with microtubule polymerization and causes mitotic arrest following apoptosis in the human prostate cancer PC-3 cell line. J Biol Chem. 2005; 280:2771–9. 10.1074/jbc.M408850200 15536083

[11] Li-Weber M. New therapeutic aspects of flavones: The anticancer properties of Scutellaria and its main active constituentsWogonin, Baicalein and Baicalin. Cancer Treat Rev. 2009; 35:57–68. 10.1016/j.ctrv.2008.09.005 19004559

[12] Thomas GM, Huganir RL. MAPK cascade signalling and synaptic plasticity. Nat Rev Neurosci. 2004; 5:173–83. 10.1038/nrn1346 14976517

[13] Raman M, Chen W, Cobb MH. Differential regulation and properties of MAPKs. Oncogene. 2007; 26:3100–12. 10.1038/sj.onc.1210392 17496909

[14] Desagher S, Osen-Sand A, Nichols A, Eskes R, Montessuit S, Lauper S, et al Bid-induced conformational change of Bax is responsible for mitochondrial cytochrome c release during apoptosis. J Cell Biol. 1999; 144:891–901. 10085289 10.1083/jcb.144.5.891 PMC2148190

[15] Luo X, Budihardjo I, Zou H, Slaughter C, Wang X. Bid, a Bcl2 interacting protein, mediates cytochrome c release from mitochondria in response to activation of cell surface death receptors. Cell. 1998; 94:481–90. 9727491 10.1016/s0092-8674(00)81589-5

[16] Wei MC, Lindsten T, Mootha VK, Weiler S, Gross A, Ashiya M, et al tBID, a membrane-targeted death ligand, oligomerizes BAK to release cytochrome c. Genes Dev. 2000; 14:2060–71. 10950869 PMC316859

[17] Rowinsky EK. The development and clinical utility of the taxane class of antimicrotubule chemotherapy agents. Annu Rev Med. 1997; 48:353–74. 10.1146/annurev.med.48.1.353 9046968

[18] Zhan Q, Antinore MJ, Wang XW, Carrier F, Smith ML, Harris CC, et al Association with Cdc2 and inhibition of Cdc2/Cyclin B1 kinase activity by the p53-regulated protein Gadd45. Oncogene. 1999; 18:2892–900. 10.1038/sj.onc.1202667 10362260

[19] Cazzalini O, Perucca P, Savio M, Necchi D, Bianchi L, Stivala LA, Interaction of p21(CDKN1A) with PCNA regulates the histone acetyltransferase activity of p300 in nucleotide excision repair. Nucleic Acids Res. 2008; 36:1713–22. 10.1093/nar/gkn014 18263614 PMC2275133

[20] Jin S, Tong T, Fan W, Fan F, Antinore MJ, Zhu X, et al GADD45-induced cell cycle G2-M arrest associates with altered subcellular distribution of cyclin B1 and is independent of p38 kinase activity. Oncogene. 2002; 21:8696–704. 10.1038/sj.onc.1206034 12483522

[21] Lu CC, Huang WS, Lee KF, Lee KC, Hsieh MC, Huang CY et al Inhibitory effect of Erinacines A on the growth of DLD-1 colorectal cancer cells is induced by generation of reactive oxygen species and activation of p70S6K and p21. J Funct Foods. 2016; 21:474–84.

[22] Liu WH, Chen YL, Chang LS. CIL-102 induces matrix metalloproteinase-2 (MMP-2)/MMP-9 down-regulation via simultaneous suppression of genetic transcription and mRNA stability. Int J Biochem Cell Biol. 2012; 44:2212–22. 10.1016/j.biocel.2012.08.021 22964005

[23] Teng CC, Kuo HC, Cheng HC, Wang TC, Sze CI. The inhibitory effect of CIL-102 on the growth of human astrocytoma cells is mediated by the generation of reactive oxygen species and induction of ERK1/2 MAPK. Toxicol Appl Pharmacol. 2012; 263:73–80. 10.1016/j.taap.2012.05.025 22683510

[24] Teng CC, Kuo HC, Sze CI. Quantitative proteomic analysis of the inhibitory effects of CIL-102 on viability and invasiveness in human glioma cells. Toxicol Appl Pharmacol. 2013; 272:579–90. 10.1016/j.taap.2013.07.009 23891858

[25] Huang WS, Chin CC, Chen CN, Kuo YH, Chen TC, Yu HR et al Stromal cell-derived factor-1/CXC receptor 4 and β1 integrin interaction regulates urokinase-type plasminogen activator expression in human colorectal cancer cells. J Cell Physiol. 2012; 227:1114–22. 10.1002/jcp.22831 21567400

[26] Chiu YW, Lin TH, Huang WS, Teng CY, Liou YS, Kuo WH et al Baicalein inhibits the migration and invasive properties of human hepatoma cells. Toxicol Appl Pharmacol. 2011; 255:316–26. 10.1016/j.taap.2011.07.008 21803068

[27] Huang WS, Kuo YH, Chin CC, Wang JY, Yu HR, Sheen JM, et al Proteomic analysis of the effects of baicalein on colorectal cancer cells. Proteomics. 2012; 12:810–9. 10.1002/pmic.201100270 22539432

[28] Kuo HC, Lu CC, Shen CH, Tung SY, Hsieh MC, Lee KC et al Hericium erinaceus mycelium and its isolated erinacine A protection from MPTP-induced neurotoxicity through the ER stress, triggering an apoptosis cascade. J Transl Med. 2016; 14:78 10.1186/s12967-016-0831-y 26988860 PMC4797317

[29] Stauffer D, Chang B, Huang J, Dunn A, Thayer M. p300/CREB-binding protein interacts with ATR and is required for the DNA replication checkpoint. J Biol Chem. 2007; 282:9678–87. 10.1074/jbc.M609261200 17272271

[30] Sarsour EH, Kumar MG, Chaudhuri L, Kalen AL, Goswami PC. Redox control of the cell cycle in health and disease. Antioxid Redox Signal. 2009; 11:2985–3011. 10.1089/ARS.2009.2513 19505186 PMC2783918

[31] Weng CJ, Yen GC. Chemopreventive effects of dietary phytochemicals against cancer invasion and metastasis: phenolic acids, monophenol, polyphenol, and their derivatives. Cancer Treat Rev. 2012; 38:76–87. 10.1016/j.ctrv.2011.03.001 21481535

[32] Jin S, Antinore MJ, Lung FD, Dong X, Zhao H, Fan F et al The GADD45 inhibition of Cdc2 kinase correlates with GADD45-mediated growth suppression. J Biol Chem. 2000; 275:16602–8. 10.1074/jbc.M000284200 10747892

[33] Hun Lee J, Shu L, Fuentes F, Su ZY, Tony Kong AN. Cancer chemoprevention by traditional chinese herbal medicine and dietary phytochemicals: targeting nrf2-mediated oxidative stress/anti-inflammatory responses, epigenetics, and cancer stem cells. J Tradit Complement Med. 2013; 3:69–79. 10.4103/2225-4110.107700 24716158 PMC3924975

[34] Dashwood RH, Ho E. Dietary histone deacetylase inhibitors: from cells to mice to man. Semin Cancer Biol. 2007; 17:363–9. 10.1016/j.semcancer.2007.04.001 17555985 PMC2737738

[35] Myzak MC, Karplus PA, Chung FL, Dashwood RH. A novel mechanism of chemoprotection by sulforaphane: inhibition of histone deacetylase. Cancer Res. 2004; 64:5767–74. 10.1158/0008-5472.CAN-04-1326 15313918

[36] Rajendran P, Ho E, Williams DE, Dashwood RH. Dietary phytochemicals, HDAC inhibition, and DNA damage/repair defects in cancer cells. Clin Epigenetics. 2011; 3:4 10.1186/1868-7083-3-4 22247744 PMC3255482

